# A *de novo* genome assembly of cultivated *Prunus persica* cv. ‘Sovetskiy’

**DOI:** 10.1371/journal.pone.0269284

**Published:** 2022-06-17

**Authors:** Maria Gladysheva-Azgari, Kristina Petrova, Svetlana Tsygankova, Irina Mitrofanova, Anatoliy Smykov, Eugenia Boulygina, Natalia Slobodova, Sergey Rastorguev, Fedor Sharko

**Affiliations:** 1 National Research Center "Kurchatov Institute", Moscow, Russia; 2 Nikita Botanical Gardens – National Scientific Centre of the Russian Academy of Sciences, Yalta, Russia; 3 Research Center of Biotechnology of the Russian Academy of Sciences, Moscow, Russia; CSIR- Institute of Himalayan Bioresource Technology, INDIA

## Abstract

Prunus persica is one of the main stone fruit crops in Crimea and southern Russia. The P. persica genome has recently been sequenced and annotated in good quality. However, for a deeper assessment of the peach genome, it is necessary to include in the research other cultivars that are in the collection of the Nikitsky Botanical Garden. The cultivars of the Nikitsky Botanical Garden are unique and differ from Western European and American ones, as they are derived from cultivars and forms originating from Central Asian, North Caucasian, Transcaucasian and Eastern European countries. In this paper, we present the assembly of the *P*. *persica* cv. ’Sovetskiy’ genome obtained using Oxford Nanopore long reads and Illumina short reads by hybrid assembly methods. The assembled genome of *P*. *persica cv*. ’Sovetskiy’ is 206.26 MB in 226 scaffolds, with N50 24 Mb, including 8 chromosomes. It contains 27140 coding genes, 26973 (99.38%) of which are annotated in at least one functional database. More than 36.05% of the genome regions were identified as repeating elements.

## Introduction

**Fig 1:**
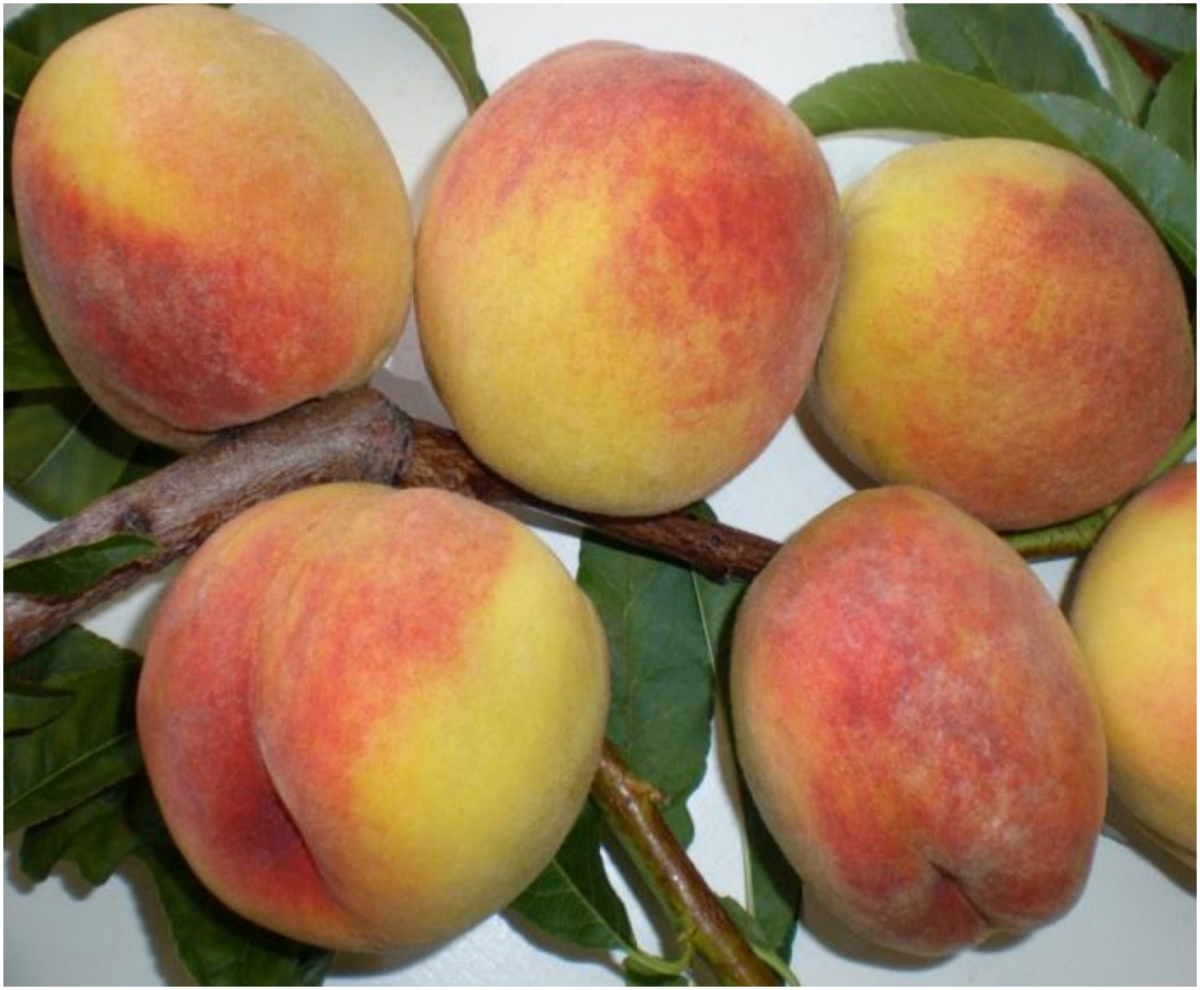
A photo of a peach fruit of *P*. *persica* cv. ‘Sovetskiy’. Photo by A. Smykov.

Common peach (*Prunus persica*) (Fig 1) is an important agricultural fruit with exceptional nutritional value [1]. New cultivars of peach are actively continuing to be created by breeders of the Nikitsky Botanical Garden. Nikitsky Botanical Garden is the largest among all botanical gardens formed on the territory of the former USSR. Over the past 30 years, more than 40 cultivars have been created that are included in the Register of industrial cultivars of Russia. Nikitsky Botanical Garden has one of the largest peach gene pools with more than 800 cultivars and forms [2]. Currently, the main task of breeders is to create cultivars of stone fruit crops that are resistant to abiotic and biotic environmental factors. In the Nikitsky Botanical Garden, long-term studies of these crops are carried out, concerning issues of biotechnology, biochemistry, physiology and reproductive biology.

Recently, genomic studies of important agricultural plants have been actively carried out all over the world [3]. These data serve as a fundamental basis for solving such important problems as: 1) identification of genes and gene networks involved in the development processes, in the adaptation of plants to conditions of abiotic and biotic stress; 2) identification of gene sets involved in the formation of economically valuable plant traits, which is necessary for carrying out work on marker-assisted selection and comparative analysis of plant genomes; 3) obtaining information about protein complexes, regulatory interactions and metabolic processes that determine the physiological and biochemical properties of a cell, organ or organism.

To date, the genome sequences of several peach cultivars with the analysis of economically valuable traits are present in the publicly available genetic databases. However, these resources do not represent the genomes of *P*. *persica* from the collection of the Nikitsky Botanical Garden, while peach cultivars from various ecological and geographical groups were involved in their creation. Therefore, these studies are quite relevant, both for further breeding and creation of new cultivars and forms, and their subsequent widespread introduction into the horticulture of the Russian Federation. In the future, these genomes can be used as reference genomes for the genotyping of the entire peach collection, including for the identification of target genes / loci responsible for resistance and adaptive properties in the conditions of southern Russia and Crimea [4].

In this work, we present a genome-wide de novo assembly of the ‘Sovetskiy’ cultivar [5], one of the first cultivars created in the USSR and belonging to the Iranian ecological-geographical group. This cultivar was bred by I.N. Ryabov in the early 50s of the last century in the Nikitsky Botanical Garden by crossing the ‘Golden Jubilee’ cultivar with fibrous pulp, belonging to the Iranian ecological-geographical group, and the Armenian ‘Narinji Late’ cultivar with cartilaginous pulp, belonging to the Northern Chinese group. The peculiarities of the ‘Sovetskiy’ cultivar include high ecological plasticity, productivity, good commercial quality of fruits, as well as high frost resistance of flower buds. The assembled genome will be a valuable resource and reference for future peach breeding, genetic improvement and comparative genomics of related species. Moreover, it will help to identify genes involved in early maturation, frost resistance, and other agriculturally significant traits.

During domestication of plants, many genes that were needed in the wild may be unnecessary for the domestic plant. Since the most suitable growing conditions were organized for this plant artificially. These allowed they not to spend unnecessary resources for growing. The definition of such needless genes can make important sense both from a practical point of view—it will be determined which genes are unimportant for cultivation and selection, and from a fundamental one—it is possible to understand how the genome changes when adapting to the artificial conditions of cultivation.

In our work, we found such genes that are "spoiled" in the ‘Sovetskiy’ cultivar and assessed the level of expression of these genes in another variety—Prunus var. Royal Glory (SRR5925639), according to the literature. The hypothesis was that: if these unnecessary genes in another variety (Royal Glory) have a low level of expression, then they are not important to the life of the plant. If these genes in another variety have a high level of expression, then they are contextual dependent—important for growing in other conditions than those in which the ‘Sovetskiy’ cultivated.

## Materials and methods

### DNA isolation, sequencing and sequence pre-processing

DNA was isolated from young leaves by the method of Lo Piccolo [6]. In the case of preparing genomic DNA for sequencing on a GridION device, purification was additionally performed on Genomic Tip 20 / G columns (Qiagen, Germany) according to the manufacturer’s standard protocol. The quality and quantity of DNA was assessed spectrophotometrically on a Nanodrop 1000 device (Thermo Scientific, USA) and using a Qubit fluorometer (Invitrogen, USA) using the Qubit ^™^ dsDNA BR Assay Kit.

To create DNA libraries, the NEBNext^®^ Ultra ^™^ II DNA Library Prep Kit for Illumina^®^ (New England BioLabs, USA) was used according to the manufacturer’s protocol. Sequencing of the obtained libraries was performed on a high-performance sequencer NovaSeq 6000 (Illumina, USA). To carry out the hybrid assembly, sequencing was carried out on a GridION device (Oxford Nanopore Technologies, UK) with a Rapid Sequencing Kit SQK-RAD004 according to the manufacturer’s recommendations.

### RNA isolation and sequence

Total RNA was isolated using the RNeasy Plant Mini Kit (Qiagen, USA) after grinding plant material (100–150 mg) in liquid nitrogen. The amount of RNA was determined using a Qubit fluorometer (Invitrogen, USA) using the Qubit ^™^ RNA HS Assay Kit. To create barcoded RNA-Seq libraries, the NEBNext^®^ Ultra ^™^ II RNA Library Prep Kit for Illumina^®^ (New England BioLabs, USA) was used according to the manufacturer’s protocol. Sequencing of the obtained libraries was performed on a high-performance sequencer NovaSeq 6000 (Illumina, USA) using the NovaSeq 6000 S1 Reagent Kit v1.5 (300 cycles).

### Genome assembly

For de novo assembly of *P*. *persica* cv. ‘Sovetskiy’, we used the hybrid de novo assembler MaSuRCA [7], which takes advantage of the high accuracy of short reads from Illumina for correcting errors in long reads from Nanopore. The mega-reads were compiled into contigs using the FLYE [8] assembler, which was designed for long reads. All MaSuRCA parameters are default values [9], for configuration parameters JF_SIZE = 4,000,000,000 and LHE_COVERAGE = 30. In addition, we did another de novo build with Canu [10] assembler, using only long reads of Nanopore. To create a more continuous assembly, these assemblies were merged using Quickmerge software [11] with contigs from Canu as input and contigs from MaSuRCA as a hybrid build. The draft assembly was polished twice and adopted the Pilon [12] algorithm v1.23 using Illumina data. All the contigs from draft assembly were anchored into chromosome-level pseudomolecules based on homology to the current peach Lovell v2.0 genome, using the RaGOO [13] program.

### Repetitive element annotation

We annotated repetitive elements of *P*. *persica* cv. ‘Sovetskiy’ with RepeatMasker v4.1.1 [14]. Repetitive elements were first identified de novo using RepeatModeler v2.0.1 [15] with parameter “-engine ncbi”. Then, the de novo database was classified using PASTEClassifier v1.0 [16] with default parameters and merged with the Repbase 20.05 database to create a repeat library as the input for RepeatMasker. We ran RepeatMasker with the parameters “-nolow -norna -engine ncbi”. To calculate the Kimura divergence [17] values and plotted the repeat landscape with repeats presented in *P*. *persica* cv. *‘Sovetskiy’* genomes we used the “calcDivergenceFromAlign.pl” script from RepeatMasker.

### Gene prediction

To predict protein-coding genes, we used the MAKER-P pipeline [18], which is designed to annotate plant genomes using three classical strategies: ab initio prediction, homology-based prediction, and transcriptome-based prediction. In the first step, we made gene prediction using Maker’s internal algorithm with transcripts and proteins, as well as genome re-masking using predicted repetitive sequences from RepeatModeler. For this, the RNA-seq reads of *P*. *persica cv*. ‘Sovetskiy’ were assembled into transcripts using Trinity [19, 20] with the paired-end option. *Prunus persica* protein sequences (GCA_000346465.2 [21]) from GenBank database were used as protein amino acids. In the second stage of Maker, we used gene models, having previously trained the Augustus v.3 [22] and Snap [23] software. To optimize the HMM search model to train Augustus and produce a trained HMM for MAKER, we applied the internal training BUSCO can perform with—long argument. In the third step of Maker, we used prediction based on RNA sequencing using GeneMarkS v4.30 [24] software and searched for tRNA using tRNAscan-SE v2.0 [25]. As a final step, we re-launched MAKER with gene prediction based on Snap and Augustus.

### Landscape of genome variations

To search for single nucleotide polymorphisms (SNPs), all reads obtained from lllumina were mapped to the reference genome of the *P*. *persica* (Lovell) using the bowtie2 [26] program, and the search for variants was performed using bcftools [27] software. The annotation of genomic variants was carried out in the nomenclature of the SnpEff program [28], in which the genomic variant is classified depending on the effect of exposure on the gene, i.e. how harmful this variant is for the gene. To search for genome structural variants (SV), such as deletion, insertion, tandem duplication, inversion and other kinds of breakends, we used the NanoVar program [29] with long Nanopore reads as input.

## Results and discussion

### Technical validation

Quantification of the DNA sample using both NanoDrop and a DNA fluorometer were performed before library construction. The 260/280 ratio of the quantified sample was 1.89, the 260/230 ratio was 1.76. The concentration of the isolated DNA was 17.1 ng/μL, estimated by the Qubit fluorometer. Gel electrophoresis revealed a single, high molecular weight DNA band with little evidence of shearing.

### DNA sequencing

After filtering and correction, the number of paired genomic reads with 150 bp length for the ‘Sovetskiy’ cultivar was 91,236,790 (27.38 Gbase). It is available at the Sequence Read Archive (SRA) of the National Center for Biotechnology Information (NCBI) under accession number SAMN19967399 [30–32]. The number of long reads obtained by GridION was 1,206,980. Low quality reads and less than 500 were filtered out. A total of 1,017,196 Nanopore reads were obtained (NCBI SRA accession number: SRR15000149 [33]). That produced 6.2 Gbase (31 × depth of the estimated genome) with average reads length of 6,157 bp and max reads length of 246,120 bp (S1 Table).

### Genome assembly and evaluation

As a result, the assembly size obtained using the MaSuRCA was 199.11 MB, and the N50 size was 668 KB. The assembly process using the Canu assembler used adjusted parameters, resulting in an assembled genome of 221.74 Mb distributed across 2,564 contigs with N50 of 307.5 kb (S2 Table). Due to the Quickmerge the N50 then improved to 2,248 kb, and the number of contigs was 465. And as a result, we obtained a total assembly size of 206.26 Mb consisting of 8 chromosomes and represented by 226 contigs with a N50 length of 24 Mb. Assembly files are deposited at GenBank [34] under the accession JAJDMZ000000000 [35]. The *P*. *persica* cv. ‘Sovetskiy’ genome assembly was evaluated with the BUSCO (version 5.1.12) [36] (benchmarking universal single-copy orthologs) searching against Eudicotsodb10 database which contains 2,326 near-universal single-copy orthologs to assess the relative completeness of genome assemblies. A total of 97.2% of the orthologs were identified as complete, 0.8% as fragmented and 2.0% as missing, indicating an overall high quality of the genome assembly (S3 Table). This criterion is a good indicator of the completeness of the Rosales genome assembly [37].

### Transposable elements

The genome of the *P*. *persica* cv. ‘Sovetskiy’ contains 36.05% of repetitive elements, among which Retrotransposons account for 7.56% of the genome and DNA transposons account for 4.51%. The largest proportion of the genome was made up of two types of repeating sequences related to long terminal repeats—Copia and Gypsy, corresponding to 4.03% and 2.51%, respectively. The number, length and percentage of each type of repeating sequences have been described (S4 Table). The content of transposable elements in the Lovell peach genome was 39.38% of the total genome and is generally similar in types (S5 Table), most likely a small difference compared to the cv. ‘Sovetskiy’ genome may be due to the redundancy of the genome (S1 Fig) [38].

### Genome annotation

In total, 27,140 genes with an average length of 2,814 bp were obtained. (S5 Table). The predicted protein-coding genes were annotated by aligning to several functional databases using blast2Go [39] software and BLAST v2.7.1+ [40] with a maximal E-value of 1e−05. As functional databases were used evolutionary genealogy of genes: Non-supervised Orthologous Groups (eggNOG) [41], SwissProt [42], Kyoto Encyclopedia of Genes and Genomes (KEGG) [43], NCBI non-redundant Nr databases [44] and Gene Ontology (GO) terms and Pfam database [45] sequences. The results showed that out of 27,140 all predicted genes were annotated: 62.83% of genes in the GO database, 41.48%, eggNOG (48.81%), KEGG (47.79%), Pfam (64.38%) and Nr (98.84%) (S6 Table). As a result of GO mapping and annotation of predicted genes sequences, a total of 255,087 GO annotations were obtained for the three categories, biological process (BP), cellular component (CC) and molecular function (MF) at a mean level of 6.84 (S2 Fig). The highest number of GO term assignments was obtained for ‘response to abscisic acid’ in the BP category. ‘Cytosol’ was the top GO term for the CC category and ‘protein binding’ was the top molecular function according to the number of identified GO terms in the MF category (S3 Fig). Most of the main annotation parameters of the P. persica cv. ‘Sovetskiy’ genome presented here were similar to the cv. ‘Lovell’ [46].

### Synteny analysis

**Fig 2:**
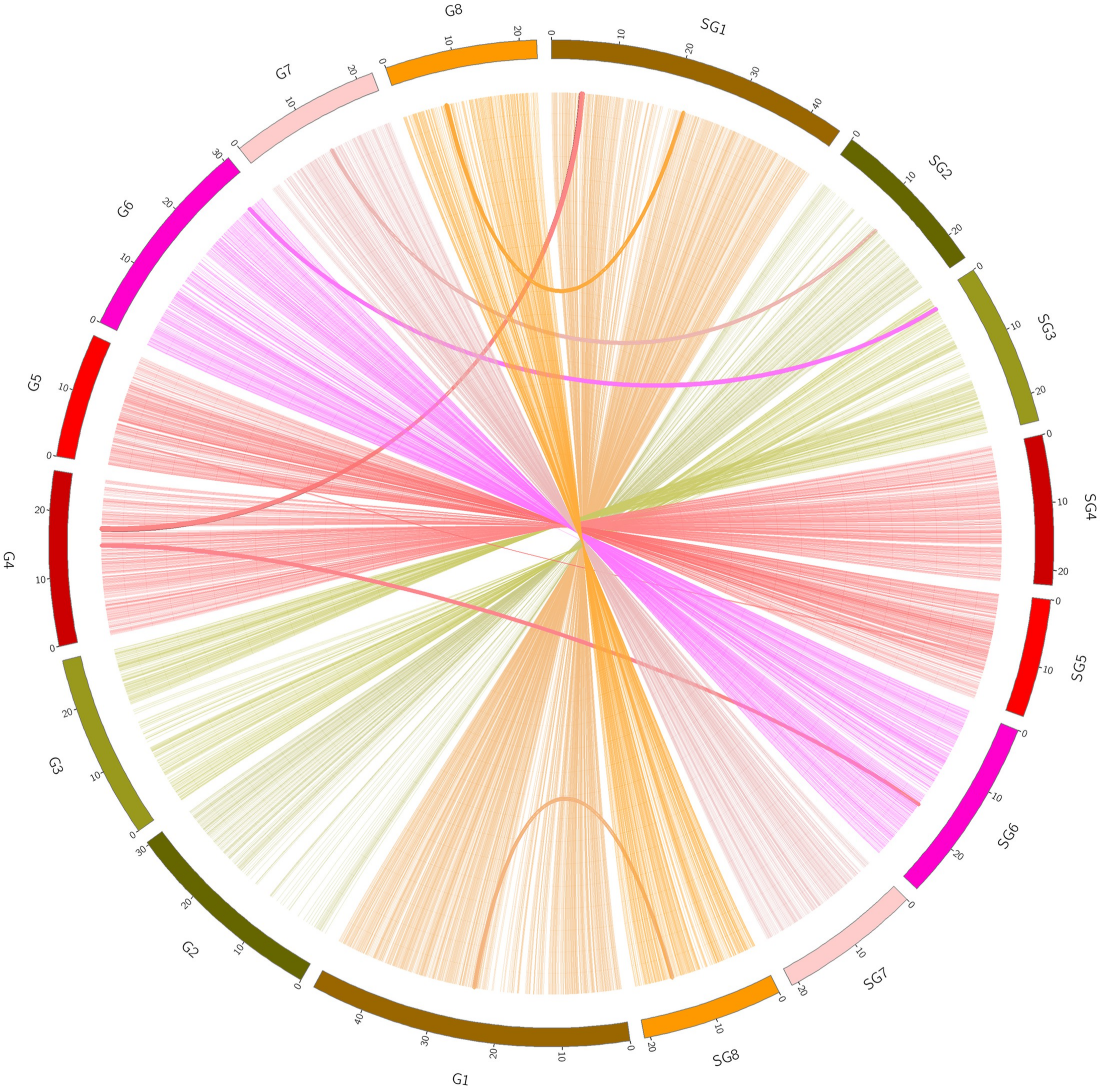
Syntenic blocks of P. persica (G1-8) and *P*. *persica* cv. ‘Sovetskiy’ *(SG1-8)*. Homologous regions of two peach genomes are connected by colored lines representing syntenic regions identified by NUCmer and mapped using Circos software.

For collinearity analysis, we compared the *P*. *persica* cv. ‘Sovetskiy’ genome with the genomes of *P*. *persica* (Lovell v2.0) using NUCmer [47] with the parameter ‘c’ is 10,000 and visualized using the Circos package [48]. Analysis of the synteny between these genomes showed that they have the same chromosomal structures and organization (Fig 2) [49]. Some synteny blocks were presented between different chromosomes, such as several regions of cv. Lovell chromosome 4 were homologous to that of pseudo-chromosomes 6 and 1 of cv. ’Sovetskiy’.

### Genome variations

In comparison with cv. ‘Lovell’ reference, 467,207 SNPs and 37,930 indels were found in total (S7 Table). We described 2,087 variants that have a high impact (i.e. completely disrupting protein structure or leading to changes in its functions), and 30,908 variants with moderate impact that potentially change protein effectiveness (S8 Table). The ratio of missense to silent SNP was 1.228 (S9 Table). As well as additional statistics of SNP, depending on their type and region are presented in S10 and S11 Tables. The SVs number for cv. ’Sovetsky’ was 20,719, of which 1,831 were insertions and 5,980 were deletions (S4 Fig) and the average length of known SVs was 2588 bp (S5 Fig). These genome variations represent the main resource of genomic variation and are known to have profound implications for phenotypic variation [50]. In plants, molecular genetic analysis revealed the functional importance of SV for protein-coding regions of genes associated with agriculturally important traits [50, 51]. Genome comparison showed that cv. ‘Sovetskiy’ and cv. ‘Lovell’ shared basically the same chromosomal structures and organization.

### Damaged genes

We selected 1110 genes based on 2,087 significant mutations in the ‘Sovetskiy’ peach genome that could negatively affect gene expression. Gene ontology analysis showed that these ‘broken’ genes were significantly enriched in molecular functions, including NAD+ nucleosidase activity and ADP binding (S6 Fig). The presence of damaged genes in the ‘Sovetskiy’ peach genome may indicate that in this cultivation environment, some genes are not beneficial in terms of selection, since the genome may provide a physiological response to a wider range of environmental influences than is present in this particular climatic zone. Or, another hypothesis, gene damage may be the result of a natural process of random mutagenesis and not be related to cultivation conditions.

To test this assumption, we obtained RNA-seq data from other peach cultivars from open databases (Prunus var. Royal Glory: SRR5925639) and looked at whether the level of expression of genes that are damaged in the ‘Sovetskiy’ cultivar differs from the level of expression of other proteins coding genes. The logic of this experiment is as follows: if genes with low expression are damaged in the ‘Sovetskiy’ cultivar, then most likely we are dealing with a random process that damages any gene. A low level of expression means that these genes are not essential for the functioning of the plant and this genetic damage is not fatal. If among the genes damaged in the ‘Sovetskiy’ cultivar there are many genes that show a high level of expression in another cultivar, then most likely we are dealing with a context-dependent process. Genes with high expression are important for another cultivar, under other cultivation conditions, but are not important for the ‘Sovetskiy’ cultivar, since the breakdown of this gene does not affect viability.

**Fig 3:**
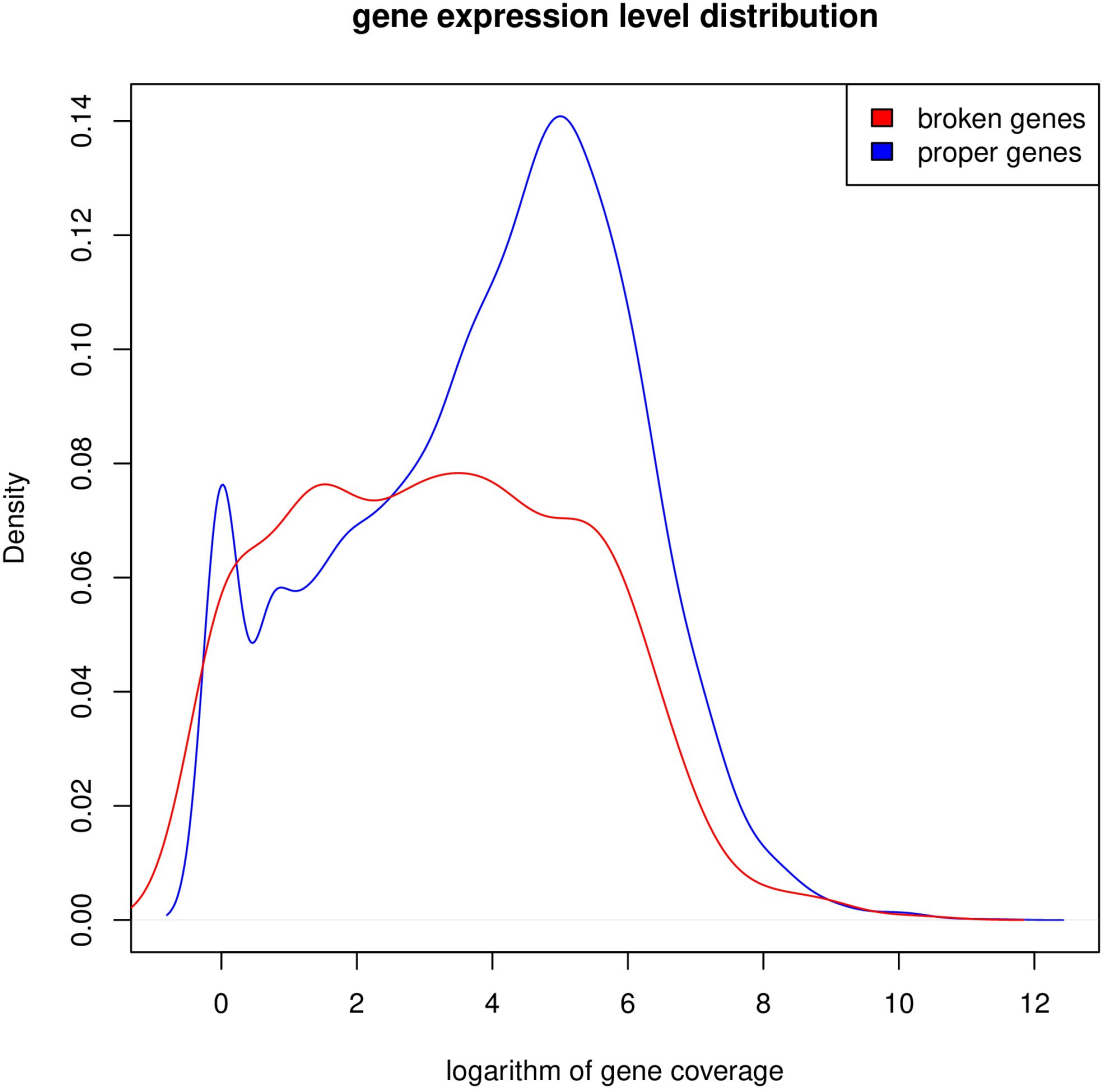
Distribution of levels of peach gene expressions according to Prunus var. Royal Glory. The expression of genes, broken in ‘Sovetskiy’ variety is shown in red colour.

The experiment showed that although the average level of expression of broken genes in Prunus var. Royal Glory is significantly lower than for the entire population of genes (mean coverage levels 127.1608 and 227.8241, respectively, pi value 0.001116), but there is a fairly large group of genes with a high level of expression (Fig 3). This suggests that our first hypothesis is more plausible–‘broken’ genes, at least a significant part of them, are context-dependent. They may be important in some cultivation conditions, but under the conditions in which the ‘Sovetskiy’ cultivar grows, their functioning is not vital.

## Supporting information

S1 Fig Landscape divergence plots in *P*. *persica* ‘Sovetskiy’. Divergences were calculated as Kimura substitution levels with adjusted CpG.
(TIF)

S2 Fig Distribution of three gene ontology (GO) category: Biological process (BP), cellular component (CC) and molecular function (MF) by GO level. (TIF)

S3 Fig Gene ontology (GO) functional classification. Distribution of annotations according to GO terms corresponding to specific GO categories: biological process, cellular components, and molecular functions.
(TIF)

S4 Fig Distribution of SV types in *P*. *persica* ‘Sovetskiy’. (TIF)

S5 Fig Size distribution of SVs across SV types in *P*. *persica* ‘Sovetskiy’. (TIF)

S6 Fig A hierarchical clustering tree summarizing the correlation among significant pathways listed of damaged genes *P*. *persica* ‘Sovetskiy’. Pathways with many shared genes are clustered together. Bigger dots indicate more significant P-values.
(TIF)

S1 Table Statistics of the different types reads of *P*. *persica* ‘Sovetskiy’. (DOCX)

S2 Table Statistics of the different methods of de novo assembly. (DOCX)

S3 Table Summary statistics derived from the BUSCO assessment of the assembled genome. (DOCX)

S4 Table Statistics of the repeated sequences. (DOCX)

S5 Table Summary statistics of all predicted genes. (DOCX)

S6 Table Statistics of gene annotation to different databases. (DOCX)

S7 Table Number variants by type. (DOCX)

S8 Table Number of effects by impact. (DOCX)

S9 Table Number of effects by functional class. (DOCX)

S10 Table Number of effects by type and region. (DOCX)

S11 Table Number of effects by type. (DOCX)
