# Supplementary figures and images for: A de novo genome assembly of cultivated Prunus persica cv. ‘Sovetskiy’
Source: PLoS One. 2022 Jun 17;17(6):e0269284. doi: 10.1371/journal.pone.0269284 (PMC9205522; doi:10.1371/journal.pone.0269284)

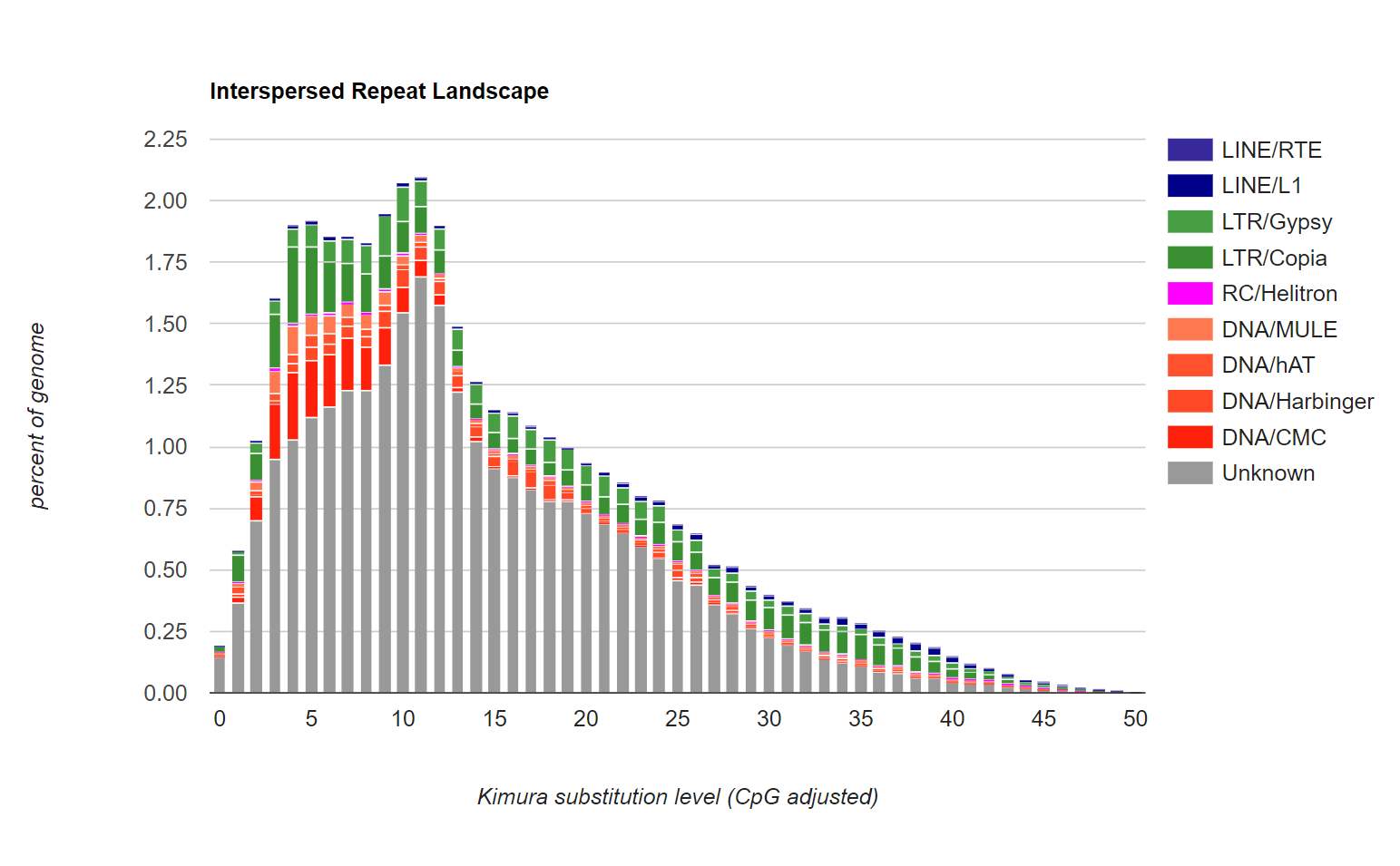

Supplement: S1 Fig — Divergences were calculated as Kimura substitution levels with adjusted CpG. (TIF) [file pone.0269284.s001.tif]

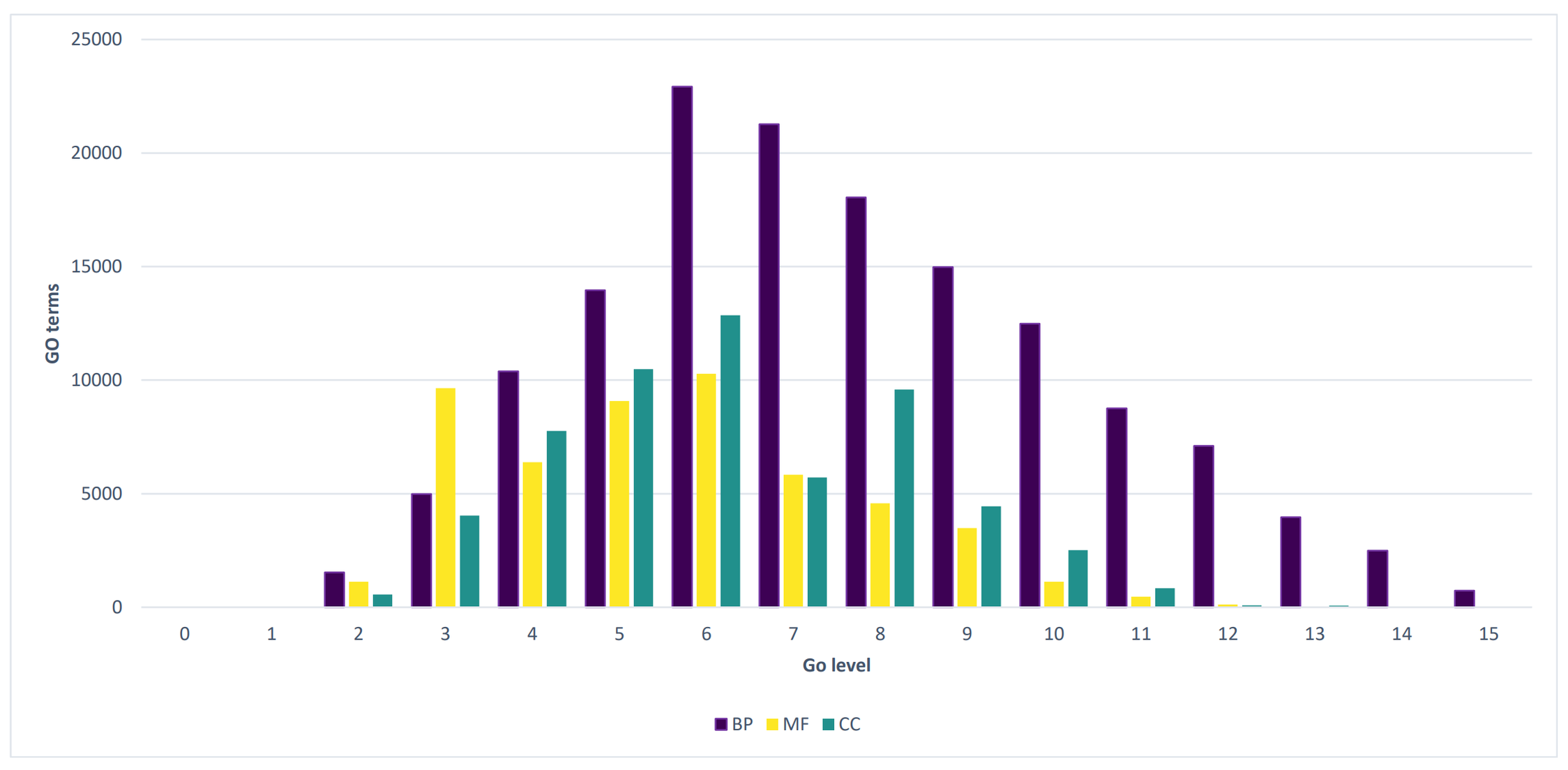

Supplement: S2 Fig — (TIF) [file pone.0269284.s002.tif]

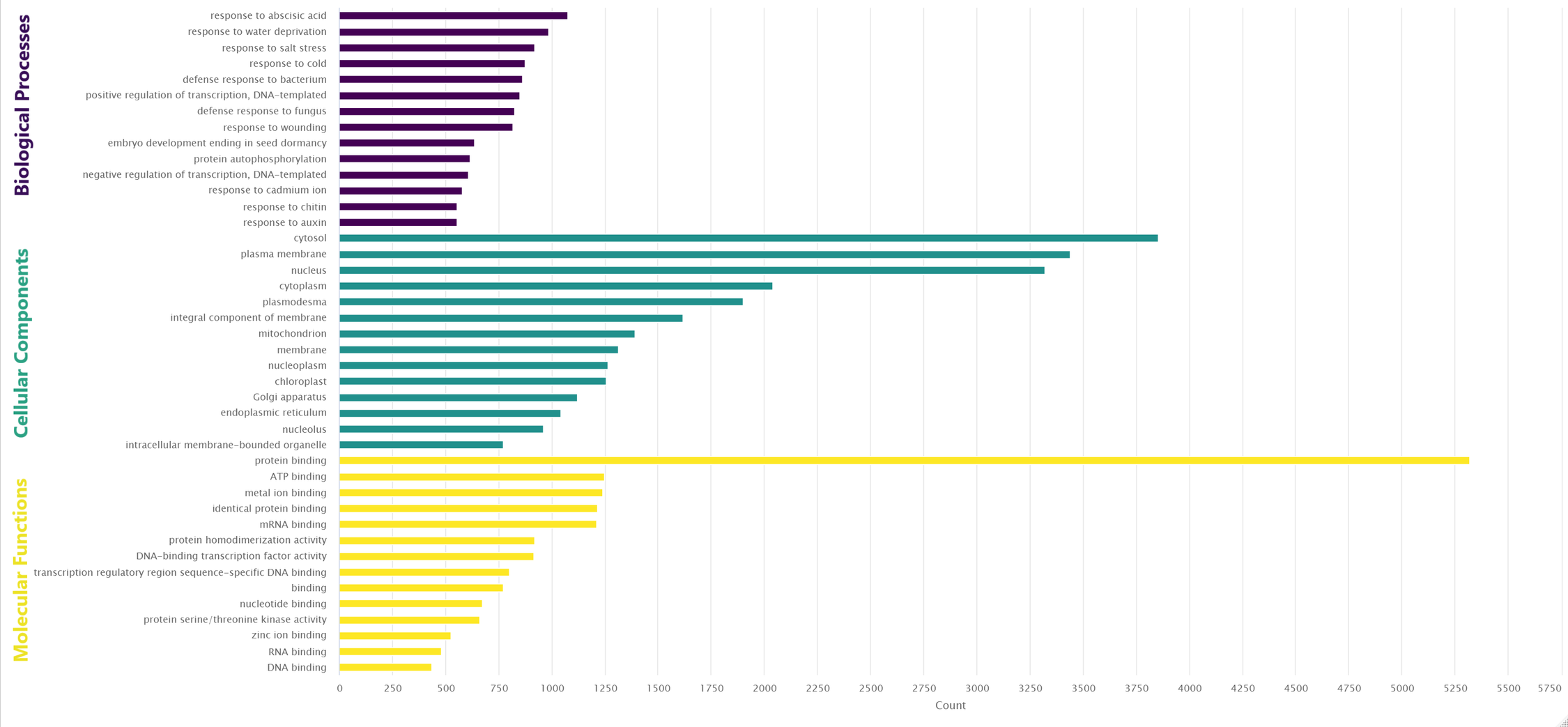

Supplement: S3 Fig — Distribution of annotations according to GO terms corresponding to specific GO categories: biological process, cellular components, and molecular functions. (TIF) [file pone.0269284.s003.tif]

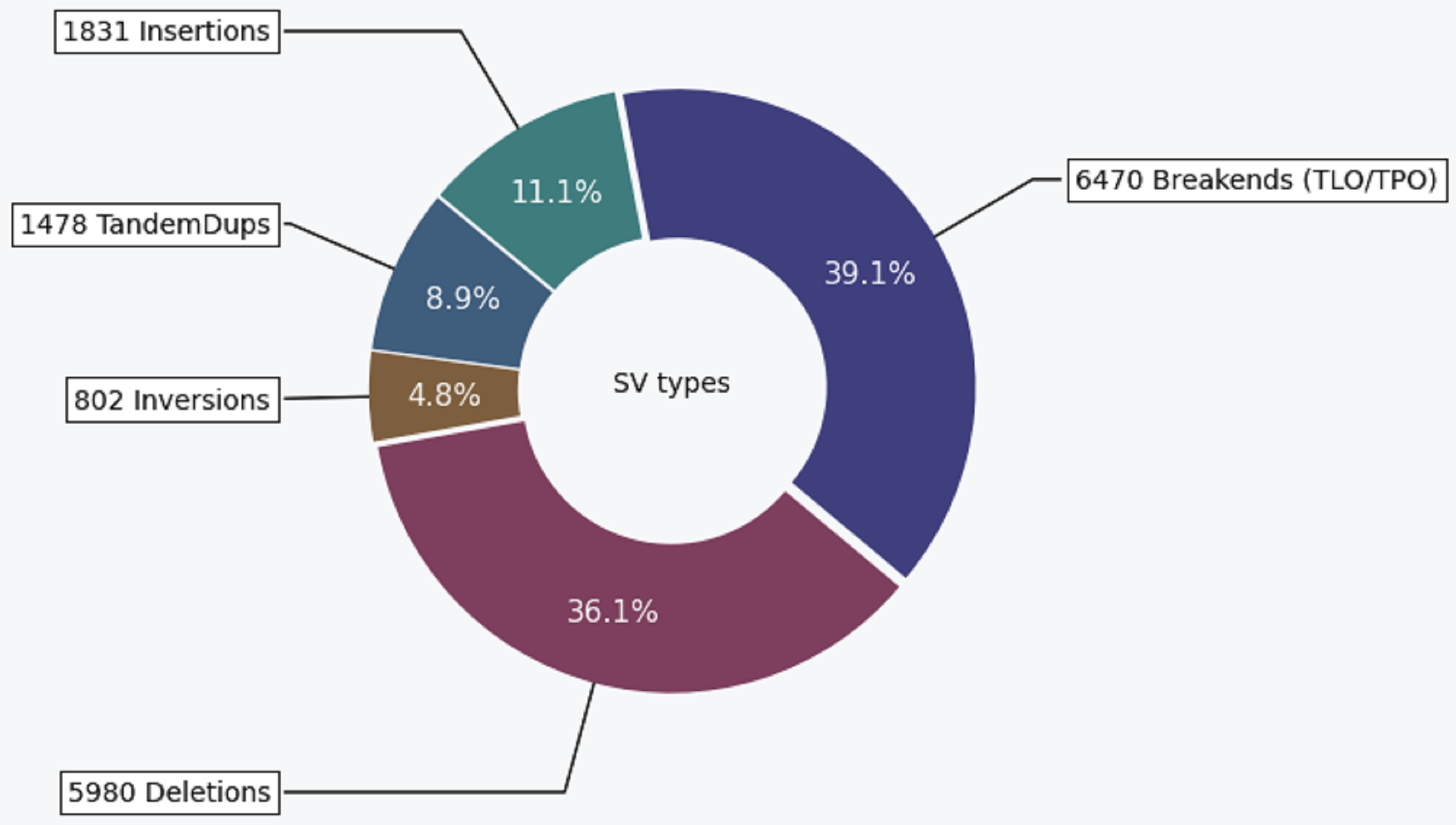

Supplement: S4 Fig — (TIF) [file pone.0269284.s004.tif]

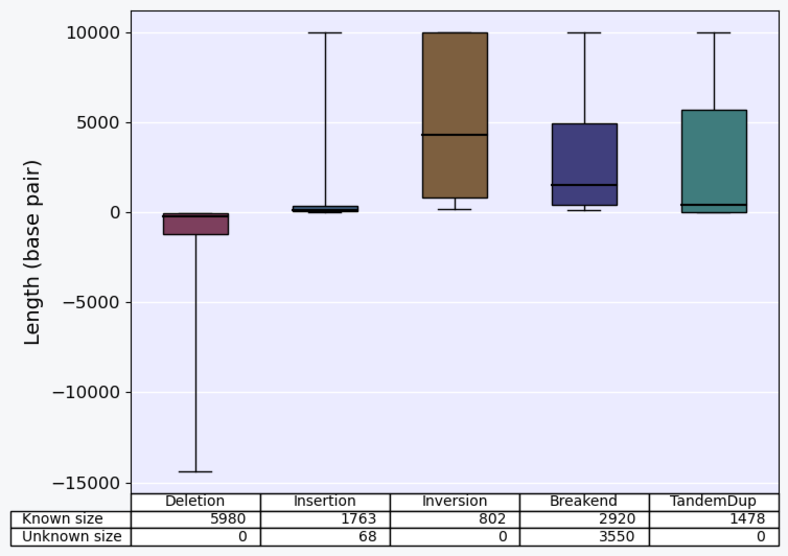

Supplement: S5 Fig — (TIF) [file pone.0269284.s005.tif]

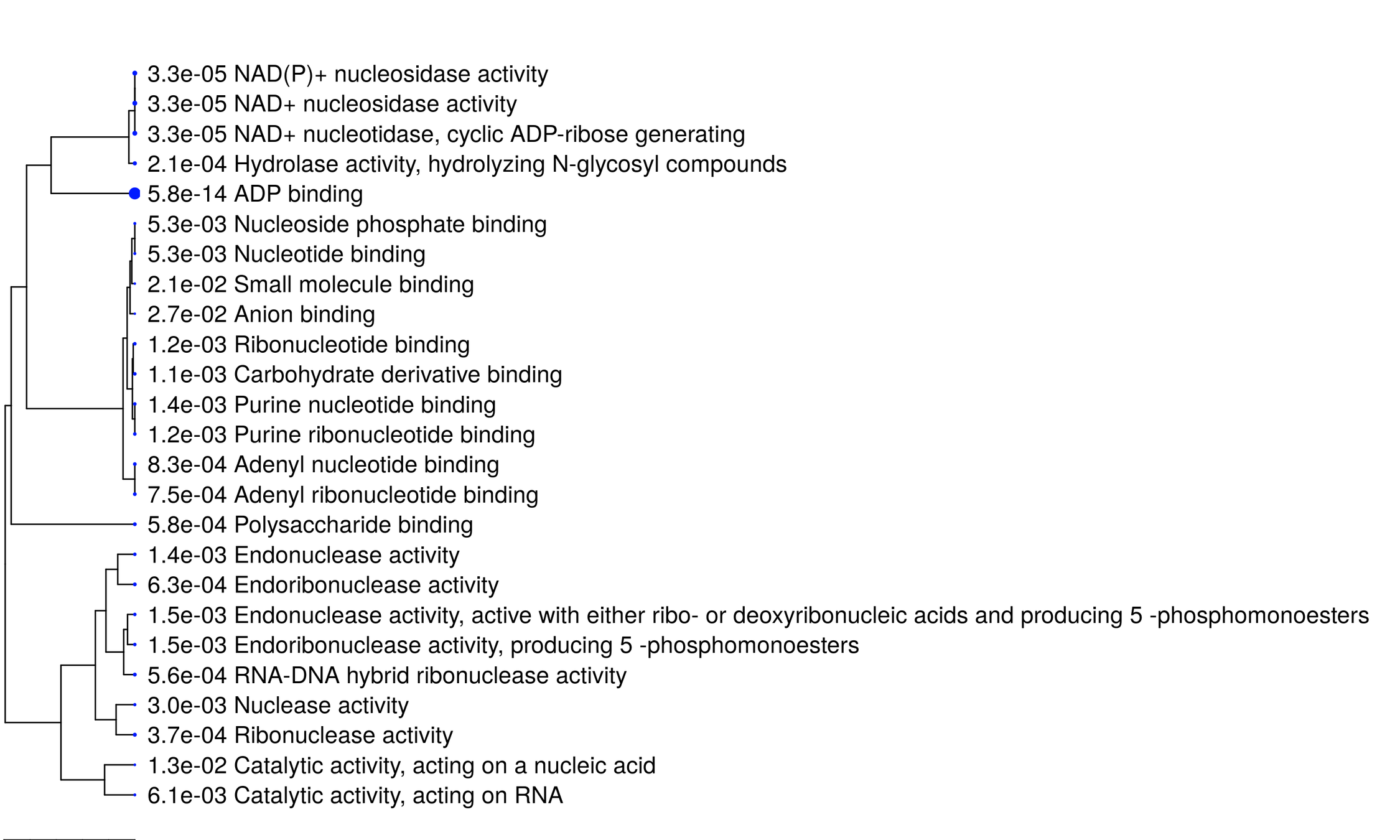

Supplement: S6 Fig — Pathways with many shared genes are clustered together. Bigger dots indicate more significant P-values. (TIF) [file pone.0269284.s006.tif]
