# Supplementary material for: A de novo genome assembly of cultivated Prunus persica cv. ‘Sovetskiy’
Source: PLoS One. 2022 Jun 17;17(6):e0269284. doi: 10.1371/journal.pone.0269284 (PMC9205522; doi:10.1371/journal.pone.0269284)
Supplement: S1 Table — (DOCX) [file pone.0269284.s007.docx]

**Table S1** Statistics of the different types reads of P. persica ‘Sovetskiy’

| Type | Number | Total length (Gb) | Average length (bp) |
| --- | --- | --- | --- |
| Illumina reads PE (150 bp) | 91,236,790 | 27.38 | 150 |
| Nanopore reads | 1,017,196 | 6.2 | 6157 |
