# Supplementary material for: A de novo genome assembly of cultivated Prunus persica cv. ‘Sovetskiy’
Source: PLoS One. 2022 Jun 17;17(6):e0269284. doi: 10.1371/journal.pone.0269284 (PMC9205522; doi:10.1371/journal.pone.0269284)
Supplement: S2 Table — (DOCX) [file pone.0269284.s008.docx]

**Table S2** Statistics of the different methods of de novo assembly

| **Methods** | **Contig**  **number** | **Contig**  **length (bp)** | **Contig**  **N50 (bp)** | **Contig**  **N90 (bp)** | **Contig**  **max (bp)** | **GC**  **content (%)** |
| --- | --- | --- | --- | --- | --- | --- |
| MaSuRCA | 1,102 | 199,119,287 | 668,591 | 100,393 | 4,001,059 | 37.35 |
| Canu | 2,564 | 221,745,929 | 307,517 | 45,070 | 4,356,979 | 37.73 |
| Quickmerge (Canu + maSuRCA) | 465 | 230,475,777 | 2,248,273 | 334,008 | 12,422,499 | 37.66 |
| Final Genome (RaGOO) | 234 | 206,269,945 | 24,048,781 | 17,576,250 | 44,979,405 | 37.35 |
