# Supplementary material for: A de novo genome assembly of cultivated Prunus persica cv. ‘Sovetskiy’
Source: PLoS One. 2022 Jun 17;17(6):e0269284. doi: 10.1371/journal.pone.0269284 (PMC9205522; doi:10.1371/journal.pone.0269284)
Supplement: S3 Table — (DOCX) [file pone.0269284.s009.docx]

**Table S3** Summary statistics derived from the BUSCO assessment of the

assembled genome

| Parameter | BUSCO groups (%) |
| --- | --- |
| Total BUSCOs | 2,326 (100) |
| Complete BUSCOs | 2,260 (97.2) |
| Complete and single-copy BUSCOs | 2,219 (95.4) |
| Complete and duplicated BUSCOs | 41 (1.8) |
| Fragmented BUSCOs | 18 (0.8) |
| Missing BUSCOs | 48 (2.0) |
