# Supplementary material for: A de novo genome assembly of cultivated Prunus persica cv. ‘Sovetskiy’
Source: PLoS One. 2022 Jun 17;17(6):e0269284. doi: 10.1371/journal.pone.0269284 (PMC9205522; doi:10.1371/journal.pone.0269284)
Supplement: S4 Table — (DOCX) [file pone.0269284.s010.docx]

**Table S4** Statistics of the repeated sequences

|  | P. persica ‘Sovetskiy’ | | | P. persica Lovell | | |
| --- | --- | --- | --- | --- | --- | --- |
| Type | Number | Length（bp） | Percentage (%) | Number | Length（bp） | Percentage (%) |
| Retroelements | 19,758 | 15,634,799 | 7.56 | 20,153 | 20,090,110 | 8.83 |
| LINE | 3,115 | 1,373,464 | 0.66 | 2,791 | 1,230,951 | 0.54 |
| LINE/L1/CIN4 | 3,064 | 1,351,116 | 0.65 | 2,791 | 1,230,951 | 0.54 |
| LINE/RTE/Bov-B | 51 | 22,348 | 0.01 | 0 | 0 | 0 |
| LTR | 16,643 | 14,261,335 | 6.90 | 17,362 | 18,859,159 | 8.29 |
| LTR/Ty1/Copia | 10,106 | 8,338,769 | 4.03 | 9,101 | 9,896,379 | 4.35 |
| LTR/Gypsy/DIRS1 | 5,765 | 5,193,330 | 2.51 | 7,505 | 7,965,550 | 3.50 |
| DNA transposons | 11,043 | 9,333,065 | 4.51 | 13,490 | 16,414,531 | 7.22 |
| DNA transposons/hobo-Activator | 3,455 | 1,370,647 | 0.66 | 4,156 | 1,640,537 | 0.72 |
| DNA transposons/Harbinger | 2,700 | 1,958,489 | 0.95 | 3,561 | 2,150,984 | 0.95 |
| Unclassified | 180,174 | 49,562,505 | 23.97 | 171,257 | 53,044,758 | 23.33 |
| Total | 210,975 | 74,530,369 | 36.05 | 206,784 | 89,549,399 | 39.38 |
