# Supplementary material for: A de novo genome assembly of cultivated Prunus persica cv. ‘Sovetskiy’
Source: PLoS One. 2022 Jun 17;17(6):e0269284. doi: 10.1371/journal.pone.0269284 (PMC9205522; doi:10.1371/journal.pone.0269284)
Supplement: S5 Table — (DOCX) [file pone.0269284.s011.docx]

**Table S5** Summary statistics of all predicted genes

| **Method** | **Gene**  **number** | **Total length (bp)** | **Average** **length (bp)** | **Average** **coding length (bp)** | **Exon length (bp)** | **Intron length (bp)** |
| --- | --- | --- | --- | --- | --- | --- |
| Maker | 27,140 | 76,373,464 | 2,814 | 1,149 | 31,188,181 | 43,584,308 |
