# Supplementary material for: A de novo genome assembly of cultivated Prunus persica cv. ‘Sovetskiy’
Source: PLoS One. 2022 Jun 17;17(6):e0269284. doi: 10.1371/journal.pone.0269284 (PMC9205522; doi:10.1371/journal.pone.0269284)
Supplement: S6 Table — (DOCX) [file pone.0269284.s012.docx]

**Table S6** Statistics of gene annotation to different databases

| Annotation database | Annotated number | Percentage (%) |
| --- | --- | --- |
| GO | 17,053 | 62.83 |
| KEGG | 12,970 | 47.79 |
| eggNOG | 11,257 | 41.48 |
| Pfam | 17,472 | 64.38 |
| SwissProt | 18,569 | 68.42 |
| Nr | 26,825 | 98.84 |
