# Supplementary material for: A de novo genome assembly of cultivated Prunus persica cv. ‘Sovetskiy’
Source: PLoS One. 2022 Jun 17;17(6):e0269284. doi: 10.1371/journal.pone.0269284 (PMC9205522; doi:10.1371/journal.pone.0269284)
Supplement: S7 Table — (DOCX) [file pone.0269284.s013.docx]

**Table S7** Number variants by type

| Type | Total |
| --- | --- |
| SNP | 467,207 |
| INS | 37,930 |
| DEL | 26,533 |
| Total | 531,670 |
