# Supplementary material for: A de novo genome assembly of cultivated Prunus persica cv. ‘Sovetskiy’
Source: PLoS One. 2022 Jun 17;17(6):e0269284. doi: 10.1371/journal.pone.0269284 (PMC9205522; doi:10.1371/journal.pone.0269284)
Supplement: S8 Table — (DOCX) [file pone.0269284.s014.docx]

**Table S8** Number of effects by impact

| Type (alphabetical order) | Count | Percent |
| --- | --- | --- |
| HIGH | 2,087 | 0.119 |
| LOW | 31,811 | 1.815 |
| MODERATE | 30,908 | 1.764 |
| MODIFIER | 1,687,464 | 96.302 |
