# Supplementary material for: A de novo genome assembly of cultivated Prunus persica cv. ‘Sovetskiy’
Source: PLoS One. 2022 Jun 17;17(6):e0269284. doi: 10.1371/journal.pone.0269284 (PMC9205522; doi:10.1371/journal.pone.0269284)
Supplement: S9 Table — (DOCX) [file pone.0269284.s015.docx]

**Table S9** Number of effects by functional class

| Type (alphabetical order) | Count | Percent |
| --- | --- | --- |
| MISSENSE | 29,817 | 54.719 |
| NONSENSE | 393 | 0.721 |
| SILENT | 24,281 | 44.56 |
| Missense / Silent ratio: 1.228 |  |  |
