# Supplementary material for: A de novo genome assembly of cultivated Prunus persica cv. ‘Sovetskiy’
Source: PLoS One. 2022 Jun 17;17(6):e0269284. doi: 10.1371/journal.pone.0269284 (PMC9205522; doi:10.1371/journal.pone.0269284)
Supplement: S10 Table — (DOCX) [file pone.0269284.s016.docx]

**Table S10** Number of effects by type and region

| Type (alphabetical order) | Count | Percent |
| --- | --- | --- |
| 3_prime_UTR_variant | 35,321 | 2.01 |
| 5_prime_UTR_premature_start_codon_gain_variant | 3,010 | 0.171 |
| 5_prime_UTR_variant | 25,916 | 1.474 |
| bidirectional_gene_fusion | 1 | 0 |
| conservative_inframe_deletion | 238 | 0.014 |
| conservative_inframe_insertion | 542 | 0.031 |
| disruptive_inframe_deletion | 205 | 0.012 |
| disruptive_inframe_insertion | 311 | 0.018 |
| downstream_gene_variant | 509,819 | 29.005 |
| feature_elongation | 3 | 0 |
| frameshift_variant | 1,104 | 0.063 |
| initiator_codon_variant | 7 | 0 |
| intergenic_region | 401,111 | 22.82 |
| intron_variant | 165,010 | 9.388 |
| missense_variant | 29,675 | 1.688 |
| non_coding_transcript_variant | 101 | 0.006 |
| splice_acceptor_variant | 153 | 0.009 |
| splice_donor_variant | 246 | 0.014 |
| splice_region_variant | 5,375 | 0.306 |
| start_lost | 107 | 0.006 |
| start_retained_variant | 8 | 0 |
| stop_gained | 472 | 0.027 |
| stop_lost | 94 | 0.005 |
| stop_retained_variant | 63 | 0.004 |
| synonymous_variant | 24,236 | 1.379 |
| upstream_gene_variant | 554,552 | 31.55 |
