# Supplementary material for: A de novo genome assembly of cultivated Prunus persica cv. ‘Sovetskiy’
Source: PLoS One. 2022 Jun 17;17(6):e0269284. doi: 10.1371/journal.pone.0269284 (PMC9205522; doi:10.1371/journal.pone.0269284)
Supplement: S11 Table — (DOCX) [file pone.0269284.s017.docx]

**Table S11** Number of effects by type

| Type (alphabetical order) | Count | Percent |
| --- | --- | --- |
| DOWNSTREAM | 509,819 | 29.095 |
| EXON | 56,574 | 3.229 |
| GENE | 1 | 0 |
| INTERGENIC | 401,111 | 22.891 |
| INTRON | 160,618 | 9.166 |
| SPLICE_SITE_ACCEPTOR | 152 | 0.009 |
| SPLICE_SITE_DONOR | 242 | 0.014 |
| SPLICE_SITE_REGION | 4,853 | 0.277 |
| TRANSCRIPT | 101 | 0.006 |
| UPSTREAM | 554,552 | 31.648 |
| UTR_3_PRIME | 35,320 | 2.016 |
| UTR_5_PRIME | 28,924 | 1.651 |
